# Impact of thrombus aspiration in frail STEMI patients

**DOI:** 10.1007/s40520-021-01848-5

**Published:** 2021-04-04

**Authors:** Pasquale Mone, Jessica Gambardella, Antonella Pansini, Mario Rizzo, Ciro Mauro, Fabio Minicucci, Gaetano Santulli

**Affiliations:** 1 University of Campania “Vanvitelli”, Piazza Miraglia, Naples, Italy; 2 ASL Avellino, Avellino, Italy; 3 University of Naples “Federico II”, Naples, Italy; 4 Einstein College of Medicine, Montefiore University Hospital, 1300 Morris Park Avenue, New York, NY 10461, USA; 5 Cardarelli Hospital, Naples, Italy

**Keywords:** Frailty, STEMI, Thrombus aspiration

## Abstract

**Background:**

Despite primary percutaneous coronary intervention (PPCI) is generally considered the best therapy in older frail adults with ST-segment elevation myocardial infarction (STEMI), the incidence of re-hospitalization for cardiovascular diseases remains significant in these patients.

**Aims:**

We hypothesized that thrombus aspiration (TA) before PPCI could be a useful treatment for reducing mortality and rehospitalizations in frail patients undergoing PPCI for STEMI.

**Methods:**

We conducted a study comparing PPCI alone *vs* TA + PPCI in frail STEMI patients. We examined a cohort of consecutive frail patients aged ≥ 65 years with first STEMI treated with PPCI between February 2008 and July 2015 at the Department of Cardiology of the “Cardarelli” Hospital in Naples, Italy.

**Results:**

The study was completed by 389 patients (PPCI: 195, TA + PPCI: 194). At 1-month follow-up, the rate of death from any cause was 7.0% in patients treated with PPCI alone vs 3.0% in patients treated with TA + PPCI (*p* 0.036), whereas death from cardiovascular causes was 6.0% in the PPCI group *vs* 3.0% in the TA + PPCI group (*p* 0.028). Equally important, the rate of re-hospitalization due to heart failure was 7.5% in the PPCI group vs 4.0% in TA + PPCI group (*p* 0.025) and the rate of re-hospitalization due to acute coronary syndrome was 10.0% in the PPCI group vs 4.5% in the TA + PPCI group (*p* 0.016).

**Conclusion:**

These results indicate the importance of TA in the treatment of STEMI in a group of high-risk patients such as elderly with frailty.

## Background

Frailty has been shown to increase the risk of cardiovascular diseases [1–6]. The CONCORDANCE registry database has reported the prevalence of frailty in older adults presenting with acute coronary syndrome (ACS) showing that at least one-third of older adults in this setting are frail, exhibiting increased morbidity, mortality, length of critical care unit stays, and readmission rates [7]. Although primary percutaneous coronary intervention (PPCI) is the best therapy in older adults with ST-segment elevation myocardial infarction (STEMI), the incidence of restenosis, heart failure (HF), re-hospitalization for ACS and death in STEMI patients remains significant and challenging [8, 9].

Thrombus aspiration (TA) before PPCI has been proposed as a useful treatment to reduce oxidative stress and distal embolization, thereby improving microvascular perfusion and reducing no-reflow phenomena [10–18]. However, randomized controlled trials (RCTs) and large trial registry studies have evidenced that TA does not significantly improve clinical outcomes in STEMI subjects [19–21]. Furthermore, some studies suggest that there is no clinical benefit in using adjunctive TA in the treatment of STEMI patients undergoing PPCI, at least in the general population [19, 20, 22]. Instead, data from the Swedish Coronary Angiography and Angioplasty Registry (SCAAR) demonstrated that TA was associated with a significantly decreased risk of stent thrombosis [23]; the SCAAR study is considered the largest cohort of consecutive STEMI patients in whom TA has been evaluated (42,829 patients: more than those in all other RCTs combined).

Nonetheless, to our knowledge there are no studies investigating the effects of TA in addition to PPCI in frail patients with STEMI.

Of note, advanced age is an important determinant of severe clinical outcomes in patients with STEMI and frailty is a strong predictor of cardiovascular events [24, 25]. Thus, we hypothesized that TA before PPCI could reduce mortality and rehospitalizations in frail patients with STEMI. To test this hypothesis, we designed a study to evaluate STEMI patients with frailty, comparing results between TA and non-TA patients, with a 30-day follow-up.

## Methods

This is an observational study investigating the relationship between TA use and outcome in addition to PPCI in frail STEMI patients. We examined consecutive patients with first STEMI treated with PPCI between February 2008 and July 2015 at the Department of Cardiology and PCI center of the “Cardarelli” Hospital in Naples, Italy. All patients with onset of symptoms < 12 h and at least 1-mm ST-segment elevation in 2 or more contiguous limb leads or at least 2 mm in two or more contiguous precordial leads or left bundle branch block underwent PPCI. Coronary angiography was performed as we previously described and validated [14, 26–28]. The culprit lesion was identified and crossed with an angioplasty guidewire. TA was performed by the operator, considering established angiographic selection criteria [29–32], followed by conventional PPCI to the culprit vessel. The thrombus grade was classified on the basis of previous studies [33, 34]:

- Grade 0 (G0), no angiographic characteristics of thrombus;
- Grade 1 (G1), possible thrombus presence;
- Grade 2 (G2), definite thrombus with largest dimension ≤ 1/2 the vessel diameter;
- Grade 3 (G3), definite thrombus, with largest linear dimension > 1/2 but < twice vessel diameter;
- Grade 4 (G4), definite thrombus, with the largest dimension ≥ 2 vessel diameters;
- Grade 5 (G5), total occlusion, unable to assess thrombus burden due to total vessel occlusion.

Inclusion criteria were: age ≥ 65 years with a confirmed frail condition (see below); presentation to the hospital for PPCI in the setting of first STEMI and feasibility of performing TA, as judged by the cardiologists. Patients with age < 65 years or non-frails, left ventricular ejection fraction < 25%, with previous myocardial infarction, revascularization, fibrinolytic therapy, or terminal cancer were excluded from the study. The investigation was designed and conducted according to the principles outlined in the Declaration of Helsinki for use of human tissue or subjects. The Institutional Review Board of University of Campania “*Luigi Vanvitelli*”, Naples, Italy approved the protocol and written informed consent was given by each patient.

Physical frailty assessment was performed right before the discharge, following the Fried Criteria [3, 35]. A diagnosis of frailty status was made in presence of at least three points out of five:

- Weight loss (unintentional loss of ≥ 4.5 kg in the past year).
- Weakness (handgrip strength in the lowest 20% quintile at baseline, adjusted for sex and body mass index).
- Exhaustion (poor endurance and energy, self-reported).
- Slowness (walking speed under the lowest quintile adjusted for sex and height).
- Low physical activity level (lowest quintile of kilocalories of physical activity during the past week).

Routine blood analyses were obtained on admission before coronary angiography. TA procedure was performed as previously described [14]. All patients (PPCI and TA + PPCI) were treated with adenosine (given 120 μg as a fast bolus followed by 2 mg in 2 min) and with bolus infusion of abciximab (0.25 mg/kg i.v. bolus); 30 days after the procedure, all patients returned to our ambulatory for follow-up. The primary outcome was all-cause and cardiovascular death. Other outcomes were re-hospitalization for ACS and HF.

### Statistical analysis

Clinical characteristics of patients were compared using the Pearson Chi square test for categorical variables and Student’s *t* test for continuous variables. Normality was assessed using the Shapiro–Wilk test. We calculated Kaplan–Meier product limits for cumulative probability of reaching an end-point and used the log-rank test for evidence of a statistically significant difference between the groups; time was measured from the first admission for the procedure until outcome. We calculated via a priori power analysis (GPOWER software) the number of patients required to reject the null hypothesis with a one-tailed type II error rate of 0.05 and a two-tailed type I error of 0.05, yielding a value of 184 participants. All calculations have been performed using the software SPSS 24.

## Results

**Fig. 1:**
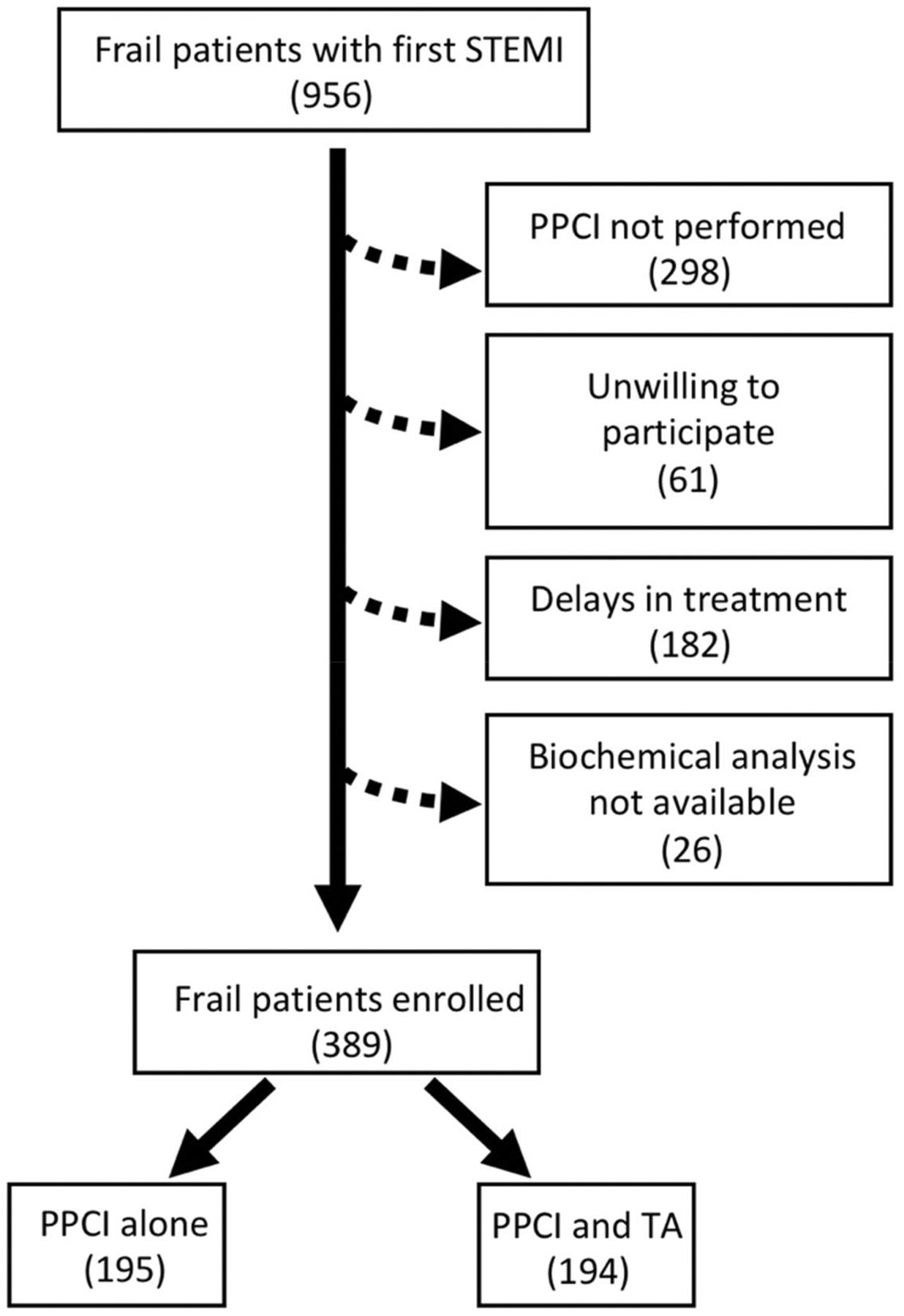
Study flow diagram

A total of 956 frail patients with suspected STEMI were admitted to the PCI center. 298 patients were excluded because PPCI was not performed, 182 patients were excluded for delays in treatment greater than 24 h, 61 patients were unwilling to provide clinical information, and biochemical analysis was not available for 26 subjects. Hence, a total of 389 patients completed the study (Fig. 1). There were no differences in the mean age, BMI, sex distribution, smoking habits, plasma cholesterol, and triglyceride levels between the two groups (Table 1).

The use of diuretics, angiotensin-converting enzyme inhibitors, beta-blockers, and calcium blockers was similar between the two groups (Table 1). Comorbidities are reported in Table 1. Angiographic data are summarized in Table 1 as well, showing that the treated lesion and the stent types were similar in the groups.

Lesion location, classification, angiographic measurements, and frequency of multi-lesion PPCI were also not significantly different between the two groups. TIMI-flow grade, corrected TIMI frame count, and myocardial blush grade pre-PPCI were similar between the two groups, whereas they were significantly improved in TA patients following PPCI; however, there was no significant difference in TIMI-flow 1 and myocardial blush grade 1 between the two groups (Table 1).

**Fig. 2:**
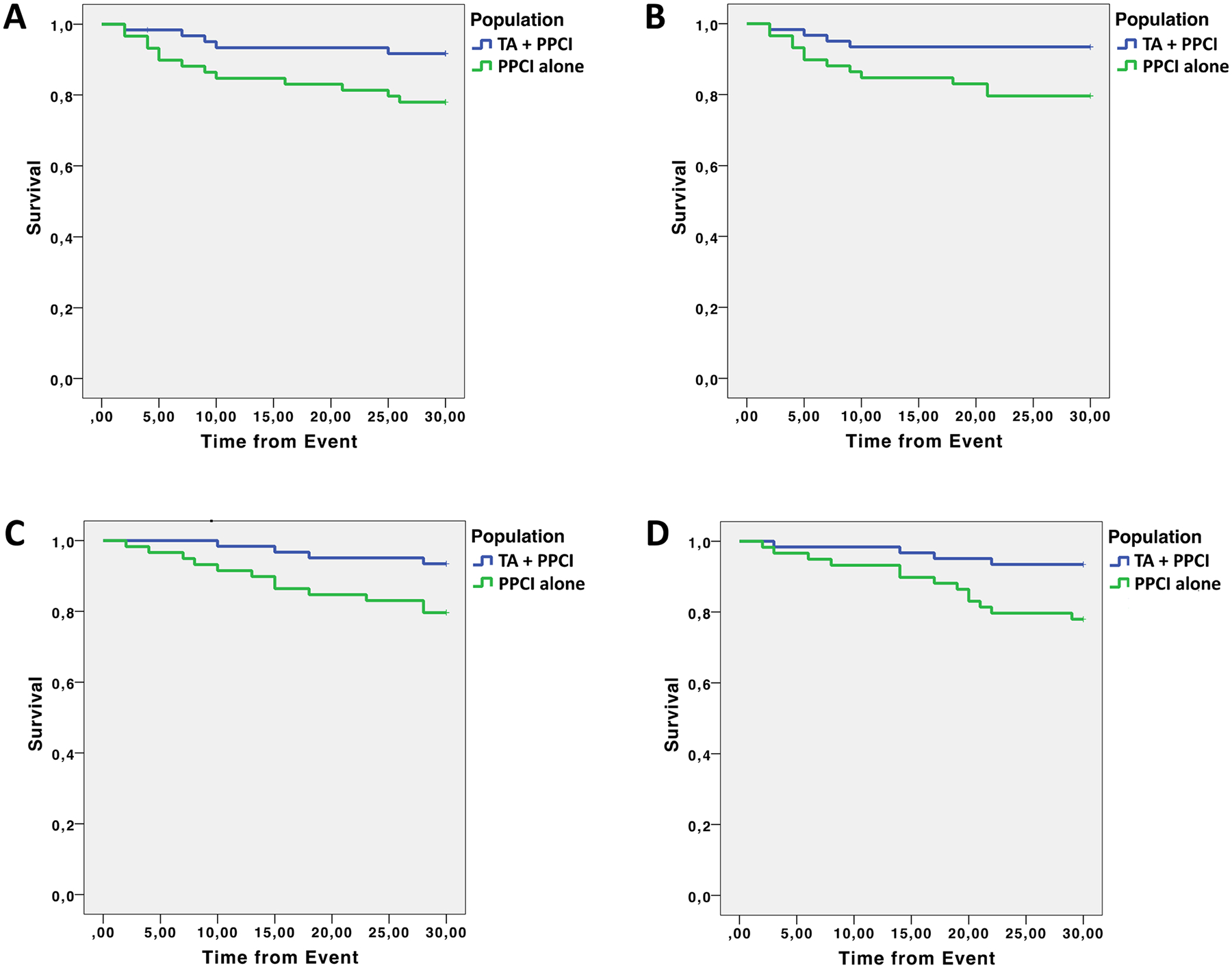
Kaplan–Meier curves for clinical outcomes: **a** death, **b** cardiac death, **c** re-hospitalization for HF, **d** re-hospitalization for ACS

Clinical outcomes At 30-day follow-up, the mortality from any cause was 7.0% in patients treated with PPCI alone *vs* 3.0% in patients treated with TA + PPCI (*p* 0.036) (Fig. 2). Similarly, death from cardiovascular causes was 6.0% in the PPCI group *vs* 3.0% in the TA + PPCI group (*p* 0.028) (Fig. 2).

The rate of re-hospitalization due to HF was 7.5% in the PPCI group vs 4.0% in TA + PPCI group (*p* 0.025) (Fig. 2). The rate of re-hospitalization due to ACS was 10.0% in the PPCI group vs 4.5% in the TA + PPCI (*p* 0.016) (Fig. 2). These results were confirmed by a multivariate analysis adjusted for age, hypertension, diabetes, and COPD.

## Discussion

The exact role of coronary TA in PPCI for STEMI is very debated [21, 23, 36–40]. Indeed, whereas early trial results encouraged this procedure, large randomized trials did not show significant clinical benefits in the general population [41, 42]. However, a recent study comparing TA + PPCI to PPCI alone evidenced lower mortality and better survival in hyperglycemic patients [14]. These results underscore the importance of TA in a group of high-risk patients such as subjects with hyperglycemia in preserving microvascular perfusion and reducing the subsequent occurrence of distal embolization and microvascular obstruction [14]. Interestingly, the presence of hyperglycemia could increase the production of oxidative stress and inflammation, responsible for the dysfunction of the microcirculation, leading to the formation of thrombi and/or atherosclerotic plaques [12].

Frail patients are known to present an intrinsic risk of cardiovascular diseases with higher mortality and higher risk of re-hospitalization [5, 7, 43]. Furthermore, they present a high oxidative stress and the oxidative damage inasmuch as the aging process itself impairs physiological functions and increases the incidence of cardiovascular diseases [13, 44–48]. Interestingly, myocardial blush grade, a parameter commonly used to assess microvascular flow [30], was better in the TA group. This finding may underline the importance of TA in protecting from distal embolization and microvascular obstruction in frailty.

The most impactful result of our study, shown by Kaplan–Meier curves, was on the hospitalization rate for ACS at 30-day follow-up (Fig. 2). Significant results were also obtained on death, cardiovascular death, as well as re-hospitalization for HF (at 30 days follow-up) (Fig. 1). Potential mechanisms underlying these observations include a reduced distal embolization and an improved microvascular perfusion; indeed, patients in the TA + PPCI group exhibited a better TIMI Flow grade compared to PPCI patients. This finding is especially striking because TA may represent a key determinant to prevent no-reflow phenomena in frail subjects and may open new fields of investigation in the management of STEMI in frail patients.

Nowadays, TA + PPCI is debated; some years ago, it was considered an important aspect of PPCI, particularly in patients with a high thrombus burden [15, 49–51]. Considering their comorbidities, frail patients could represent a sub-class in which TA + PPCI may have a great impact.

Several limitations of the present study warrant consideration. First, the follow-up period of 30 days is short; nevertheless, observing significant differences between PPCI and TA + PPCI in several outcomes already after 1 month is noteworthy, especially in a population of elderly patients. Second, the sample size of our two groups is relatively small; however, as mentioned in “Methods”, we had performed an a priori power analysis, based on our preliminary data, showing that the estimated sample size was 184 patients. We did not evaluate the neurological and cerebrovascular outcomes in our population, which remain a controversial point of TA: indeed, whereas an increased risk of stroke has been reported in the Trial of Routine Aspiration Thrombectomy with PCI versus PCI Alone in Patients with STEMI (TOTAL) [19], such a finding has not been confirmed in the Thrombus Aspiration in ST-Elevation Myocardial Infarction in Scandinavia (TASTE) [21] or in the SCAAR [23] studies. Further studies, ideally randomized trials focused on elderly populations, with longer follow-up and larger cohorts, are required to confirm our results.

## Conclusions

Taken together, our data suggest that in the treatment of STEMI, adding TA to PPCI is associated with a significant improvement in 30-day mortality and hospitalizations due to HF and ACS in frail elderly patients, compared to conventional PPCI alone.

## Figures and Tables

**Table 1:** Baseline and follow-up clinical characteristics, angiographic and procedural data

	PPCI		TA + PPCI	
	Baseline *n*195	Follow-up *n* 181	Baseline *n* 194	Follow-up *n* 188
Age (years)	72 ± 5.5	/	71.5 ± 5.0	/
Female sex, *n* (%)	104	/	102	/
BMI (kg/m^2^)	28.1 ± 1.7	27.2 ± 1.8	28.3 ± 1.6	26.6 ± 1.8
SBP (mmHg)	131.2 ± 10.5	127.2 ± 9.0*	131.4 ± 9.0	125.3 ± 8.5*
DBP (mmHg)	78.6 ± 6.6	76.3 ± 6.5	79.2 ± 6.8	76.3 ± 6.8
Heart rate (bpm)	87.1 ± 7.9	76.1 ± 6.9*	87.0 ± 9.4	75.2 ± 9.3*
Cigarette smoking, n (%)	81 (41.5)		79 (41.0)	
TIMI flow grade pre				
Grade 0, *n* (%)	86 (44.0)		87 (46.0)	
Grade 1, *n* (%)	16 (8.0)		13 (7.0)	
Grade 2/3, *n* (%)	93 (48.0)		92 (47.0)	
TIMI flow grade post				
Grade 0, *n* (%)	11 (5.5)		3 (1.5)**	
Grade 1, *n* (%)	58 (30.0)		49 (25.5)	
Grade 2/3, *n* (%)	126 (64.5)		142 (73.0)**	
Myocardial blush grade pre				
Grade 0, *n* (%)	82 (42.0)		89 (46.0)	
Grade 1, *n* (%)	14 (7.0)		10 (5.0)	
Grade 2/3, *n* (%)	99 (51.0)		95 (49.0)	
Myocardial blush grade post				
Grade 0, *n* (%)	16 (8.0)		5 (3.0)**	
Grade 1, *n* (%)	59 (30.5)		44 (22.5)	
Grade 2/3, *n* (%)	120 (61.5)		145 (74.5)**	
Corrected TIMI frame count pre	83.4 ± 24.1		87.8 ± 23.3	
Corrected TIMI frame count post	31.1 ± 23.8		24.7 ± 19.2**	
Killip class				
Class 1, *n* (%)	59 (30.5)		56 (29)	
Class 2, *n* (%)	51 (26)		62 (32)	
Class 3, *n* (%)	82 (42.0)		72 (37)	
Class 4, *n* (%)	3 (1.5)		4 (2)	
Thrombus grade				
G0 none	16 (8.0)		18 (9.0)	
G1 possible	27 (14.0)		25 (13.0)	
G2 small	20 (10.0)		23 (12.0)	
G3 medium	33 (17.0)		29 (15.0)	
G4 large	37 (19.0)		35 (18.0)	
G5 vessel occlusion	62 (32.0)		64 (33.0)	
Comorbidities				
Diabetes, *n* (%)	121 (62.0)		122 (61.0)	
Hypertension, *n* (%)	138 (70.0)		134 (68.0)	
Dyslipidemia, *n* (%)	90 (46.0)		88 (45.0)	
Prior Stroke, *n* (%)	16 (8.5)		15 (8.0)	
Cerebrovascular disease, *n* (%)	32 (17.0)		30 (16.5)	
Chronic lung disease, *n* (%)	26 (14.0)		27 (15)	
Active treatments				
β-Blockers, *n* (%)	137 (70)	174 (89.0)*	134 (68.0)	178 (89.0)*
ACE inhibitors, *n* (%)	104 (53.0)	106 (54.0)	105 (54.0)	114 (57.0)
Angiotensin receptor blockers, *n* (%)	30 (16.0)	40 (20.0)	33 (17.5)	45 (22.5)
Calcium inhibitors, *n* (%)	42 (21.5)	59 (29.5)	48 (24.0)	55 (27.5)
Nitrate, *n* (%)	/	156 (79.0)	/	167 (85.0)
Statins, *n* (%)	54 (27.5)	195 (98.5)*	55 (27.5)	191 (99.0)*
Diuretic, *n* (%)	16 (8.5)	49 (24.5)*	16 (8.0)	43 (21.5)*
Insulin, *n* (%)	34 (17.5)	45 (22.5)	35 (17.5)	47 (23.0)
Oral antidiabetic, *n* (%)	48 (24.5)	80 (40.0)*	45 (23.0)	89 (44.5)*
Aspirin, *n* (%)	49 (25.0)	192 (99.0)*	44 (22.5)	191 (99.0)
Thienopyridine, *n* (%)				
Dual anti-platelet therapy, *n* (%)	/	183 (91.5)	/	193 (96.5)***
Low-molecular weight heparin, *n* (%)	/	14 (7.0)	/	28 (14.0)***
Vitamin-K antagonist, *n* (%)	/	12 (6.0)	/	11 (5.5)
Laboratory parameters				
Plasma glucose (mg/dl)	191.1 ± 22.7	126.4 ± 23.3*	190.3 ± 20.2	121.2 ± 21.3*,***
Cholesterol (mg/dl)	205.2 ± 20.4	202.2 ± 20.4	204.6 ± 22.6	192.8 ± 24.7***
LDL-cholesterol (mg/dl)	132.7.9 ± 17.6	128.2 ± 20.1	131.1 ± 21.5	122.8 ± 25.1*,***
HDL-cholesterol (mg/dl)	38.2 ± 6.4	42.2 ± 3.4	37.1 ± 3.5	38.9 ± 3.5***
Triglycerides (mg/dl)	181.0 ± 19.1	159.0 ± 19.1*	185.0 ± 24.0**	145.6 ± 31.1*,***
Creatinine (mg/dl)	1.0 ± 0.1	1.0 ± 0.1	1.0 ± 0.1	1.0 ± 0.1
cTnT (ng/l)	5.4 ± 1.5	/	5.6 ± 1.4	/
Angiography data				
Number of diseased vessels				
1-VD, *n* (%)	150 (75.5)	/	153 (78.0)	/
2-VD, *n* (%)	43 (23.5)	/	40 (21.5)	/
3-VD, *n* (%)	2 (1.0)	/	1 (0.5)	/
Lesion location				
RCA, *n* (%)	61 (31.5)	/	68 (35.0)	/
LAD, *n* (%)	86 (44.0)	/	83 (42.5)	/
LM, *n* (%)	7 (3.5)	/	8 (4.0)	/
LCx, *n* (%)	41 (21.0)	/	35 (18.5)	/
LVEF				
> 50%, *n* (%)	108 (55.0)	129 (65.5)	112 (57.0)	142 (72.5)*
41–50%, *n* (%)	59 (35.5)	59 (31.0)	52 (27.5)**	49 (26.0)
25–40%, *n* (%)	28 (14.5)	7 (3.5)*	30 (15.5)	3 (1.5)*
Stent type				
DES, *n* (%)	170 (86.0)	/	176 (90.0)	/
BMS, *n* (%)	25 (14.0)	/	18 (10.0)	/
Multivessel intervention, *n* (%)	49 (24.5)	/	44 (22.0)	/

Data are mean ± SD or *n* (%)

*1-VD* single-vessel disease, *2-VD* two-vessel disease, *3-VD* three-vessel disease, *BMS* bare metal stent, *DBP* diastolic blood pressure, *DES* drug-eluting stent, *LAD* left anterior descending, *LCx* left circumflex artery, *LM* left main, *LVEF* left ventricular ejection fraction, *MLD* minimum luminal diameter, *PPCI* primary percutaneous coronary intervention, *RCA* right coronary artery, *SBP* systolic blood pressure, *TA* thrombus aspiration

The symbol * is indicating a *p* < 0.05 with the comparison of baseline vs. follow-up; the symbol ** is indicating the *p* < 0.05 with the comparison of baseline PPCI vs. Baseline TA + PPCI; the symbol *** is indicating the *p* < 0.05 with the comparison of follow-up PPCI vs. TA + PPCI
